# What Drives Patients Affected by Depression to Share in Online Depression Communities? A Social Capital Perspective

**DOI:** 10.3390/healthcare7040133

**Published:** 2019-11-04

**Authors:** Yingjie Lu, Taotao Pan, Shasha Deng

**Affiliations:** 1 School of Economics and Management, Beijing University of Chemical Technology, Beijing 100029, China; 2 School of Business and Management, Shanghai International Studies University, Shanghai 200083, China

**Keywords:** online depression community, social capital, information sharing

## Abstract

Online depression communities give people additional opportunities to share their experiences and exchange social support to care for themselves in fighting against depression. We aimed to explore what drives patients to share in online depression communities. We used three dimensions of social capital (structural, relational, and cognitive) to explain their sharing behaviors. We further proposed that five factors (social interaction ties, a sense of shared identity, trust, expertise, and a sense of shared values) will have significant, positive effects on sharing behaviors and that there are differences among patients who have spent different lengths of time participating in online depression communities. We then chose a popular online depression community in China as our data source and obtained a dataset consisting of 31,440 posts from 197 members. Then, we employed panel data regression analyses to test all six hypotheses. The results revealed that all five factors had significant, positive effects (*p* < 0.01) on patients’ sharing behaviors, and the effects were significantly different across groups. Our empirical results help designers and managers of online depression communities take specific measures to facilitate community members’ access to social capital resources. Meanwhile, our results have implications for existing health management and e-health literature.

## 1. Introduction

### 1.1. Background

In recent years, mental health problems, such as depression, anxiety, and bipolar disorder, have become more common. Among the various mental problems, depression is increasingly prevalent and has become a serious public concern. A recent report from the World Health Organization found that the number of people with depression increased by 18% from 2005 to 2015, and now there are approximately 300 million people worldwide suffering from depression [1]. Depression is considered to be associated with a range of negative outcomes, including cognitive impairment, substance abuse, self-harm, and suicide [2]. A number of studies have indicated that social support is widely considered to be protective against depression [3], and a lack of social support presents a greater risk for depression. Social support may come from diverse sources (e.g., parents, relatives, friends, neighbors) [4], which can have different positive impacts on the treatment of depression. It is worth noting that peer support is considered to be a strongly preventive strategy for depression. Peers can share experiences, offer advice on condition management [5], provide emotional support, and provide valuable information [6].

However, public stigma held by others leads to stigmatizing attitudes, prejudices, and actions towards patients struggling with depression. Stigmatized patients may internalize perceived prejudices, develop negative feelings about themselves, and are thus more likely to self-stigmatize. They feel shame and embarrassment about having a mental illness, and these feelings limit social interactions with others and increase their social isolation [7]. Under these conditions, social media, especially online health communities, have become increasingly recognized as a promising platform for communication about mental health issues [8]. In a social study [9], up to 41% of participants with depression stated that online communities helped them reconnect with individuals and overcome their depressive states. The emerging online depression community has attracted wide attention. For example, one research team [10] investigated 25 popular online depression communities worldwide to explore the potential benefits these communities offer people with depression. Some famous social media platforms have included depression forums since depression is one of the most frequently discussed health issues. For example, a survey on Reddit [11], a popular content-sharing social networking site, found that the depression community is one of the most active online communities, and it has been active for 7 years with 145,821 subscribers. Another study [12] collected data from a large online community and found more than 12,000 active members have made more than 40,000 posts and approximately 180,000 comments related to depression from 2000 to 2014. Some other recent studies have investigated depression in some popular social media platforms, including Twitter [13] and Facebook [14]. This phenomenon is also observed in many developing countries such as China. An estimated over 100 million Chinese people have a diagnosable mental disorder, of whom 91% have never received any treatment [15]. Many of them with major depressive disorder have sought information and support online in recent years [16]. For example, some popular Chinese online health communities, such as DXY (dxy.cn) and tieba (tieba.baidu.com), consist of depression discussion boards and have attracted over millions of patients with depression involved in online discussions. Another survey [17] on the website Sunshine, one of the most popular online depression communities in China, found that it had 161,211 registered members and over 990,000 posts by August 2017.

Online depression communities give people additional opportunities to make friends, share their experiences, and exchange social support to care for themselves in their fight against depression. Such online communities can provide “just in time” support, overcome geographic barriers, facilitate the open discussion of health concerns [18], and reduce social isolation by providing opportunities for sharing information and providing practical advice. In addition, the anonymous nature of online communities makes participants, particularly those dealing with health-related stigmatization, feel safe.

Despite the important benefits of online communities to members with depression, there is a critical issue that remains unsolved concerning the information shared among the members involved in online depression communities. As we know, the key determinant of a successful online community is the active participation of its members in community activities to create and share information [19]. However, the information is primarily user-generated on a voluntary basis and is generally free. Community members do not have an obligation to create public goods even though they may have the need to consume such goods. If there is insufficient information provided by online communities, the support that the member feels will inevitably decline, leading to growing dissatisfaction with the online communities. The problem is even more severe in online depression communities. The aim of user participation in online depression communities is to seek peer support to help alleviate symptoms of depression, so the members will inevitably feel disappointed and are highly likely to leave if peer support in online communities is lacking. Therefore, it has become an urgent issue to determine what drives users to share information in an online depression community.

Some prior studies have attempted to explore the key antecedents of information sharing in online health communities. The studies have pointed out that members in online health communities primarily desire to gain nonmonetary, rather than monetary, benefits, which is different from other types of virtual communities where people benefit by gaining monetary rewards or enjoyment [20,21]. Thus, some extrinsic motivations (e.g., monetary reward and enjoyment) may not be the main drivers of information sharing in online health communities [22]. Some scholars have proposed that members in online health communities are intrinsically motivated to share information in order to help others [23,24]. For example, altruism could be an important knowledge-sharing motivation [23]. Furthermore, users may share information out of their sympathy for others who suffer from the disease [25]. Although many studies have emphasized the significance of the motivation to share information in online health communities [26,27,28], the influence of motivation on information sharing in online depression communities has been rarely examined. For patients with major depression, peer support through information sharing among members is extremely significant in improving depression symptoms [3]. Therefore, a comprehensive understanding of what motivates information sharing in the context of online depression communities is of great significance for attracting more members to share information and provide social support in preventing depression.

Over the years, scholars have developed some theories and constructs to better understand information sharing in virtual communities [27,28], among which social capital theory is particularly well suited to explain the paradox of why community members voluntarily share information without any immediate benefits. Social capital theory is one of the most influential theories that evolved from new economic sociology. Nahapiet and Ghoshal classified social capital into three dimensions: structural, relational, and cognitive [29]. Their study found that three forms of social capital were closely associated with information sharing. We can postulate that social capital theory also applies to online depression communities because they are a special type of virtual community. Therefore, in this study, we have attempted to use social capital theory to explore what drives patients to share information in an online depression community. In addition, patients are likely to change their perception of an online depression community as they take more time to participate in the online community, resulting in some dimensions of social capital having various important roles in the information-sharing decisions of participants. We are also concerned with the issue of whether social capital has a different effect on information sharing for those patients who have spent different lengths of time participating in an online depression community. Thus, in our study, we attempted to investigate the following research questions:

RQ1: What drives patients affected by depression to share information in an online depression community from the perspective of social capital?

RQ2: Are there differences concerning the effect of social capital on information sharing for patients who have spent different lengths of time participating in an online depression community?

### 1.2. Theoretical Background and Research Hypotheses

#### 1.2.1. Information Sharing

Information sharing has always been a hot issue in the study of knowledge management. It seems paradoxical that sharing knowledge eventually causes the possessor of knowledge to lose his or her unique value relative to what others know [30], which benefits all participants except for the contributor [31]. The dilemma also occurs in the context of online communities. Community members can retrieve information without contributing anything [32]; conversely, information sharers must invest a great deal of time and effort to contribute information without receiving any personal benefit. If every member in an online community chooses to free-ride and nobody shares information, the online community will cease to exist, and nobody receives any benefit at all. In the last few years, much research has been conducted on possible solutions to such a dilemma. It is widely considered that the lack of sufficient rewards and benefits to compensate individuals for the costs of sharing knowledge becomes a common barrier to knowledge sharing [33,34,35]. However, it is difficult, or even impossible, in the context of online depression communities to solve the information-sharing dilemma through rewards. On the one hand, the reasons that patients participate in online depression communities vary depending on individual goals; thus, information sharers cannot anticipate how necessary their contributions are. On the other hand, it is difficult to measure and evaluate the value of patients’ contributions since participation in online depression communities is usually voluntary and anonymous.

#### 1.2.2. Social Capital Theory

Social capital theory could help explain why members in online communities choose not to free-ride and suggest that community members forego the tendency to free-ride due to the influence of social capital. Social capital was first presented by Nahapiet and Ghoshal as an integrative framework for understanding the creation and sharing of knowledge in organizations. Social capital is defined as “the sum of the actual and potential resources embedded within, available through, and derived from the network of relationships possessed by an individual or social unit” [29]. According to Nahapiet and Ghoshal’s study, social capital can be classified into three distinct dimensions: structural, relational, and cognitive. Structural capital is defined as the overall pattern of connections between network actors and represents the configuration of impersonal relationships. Relational capital describes the kind of personal relationships people have developed with each other through a history of interactions [36], primarily characterized by trust, the norm of reciprocity, and identification [26]. Cognitive capital refers to those resources that provide shared representations, interpretations, and meaning among members [37].

While most previous studies of social capital have mainly focused on its impact on organization-level outcomes, there have been a few studies that have tried to examine the relationship between social capital and information sharing at the individual level, with some evidence suggesting that social capital has a significant influence on individuals’ information sharing. Furthermore, some recent studies have applied social capital theory to the context of online virtual communities and explored whether social capital theory can help explain why community members are willing to share information without extrinsic benefits [37]. The results have provided support for the argument that social capital is a significant predictor of individual knowledge contribution in online virtual communities. Therefore, we believe that social capital theory also applies to online depression communities because they are a special type of virtual community.

#### 1.2.3. Research Hypotheses

Following the theoretical framework of social capital, this study developed research hypotheses to examine how three dimensions of social capital (structural, relational, and cognitive) relate to information sharing in online depression communities and then attempted to find what drives users to share information in an online depression community.

The structural dimension of social capital refers to the pattern of connections between individuals in a network. Prior research has focused on structural capital at the organizational level and assessed the network density of the overall organization [29]. The findings suggested that a dense network with a large number of strong ties among its members could help information exchange in organizations. Some subsequent studies adapted structural capital to the individual level and considered how structural capital relates to an individual’s ability to make strong ties with others in a network [26]. An individual with a relatively high proportion of direct ties to other members is considered to be centrally embedded in the network [38]. Some research has pointed out that an individual’s position in the network influences his or her willingness to contribute knowledge to others [26]. The closer an individual is to the center of the network, the more he/she is willing to share information with others. This could well be applied to the context of online depression communities. Patients with depression share the topics that interest them by posting and replying to others’ postings, thus creating many social ties between members. Patients with a large number of strong social interaction ties are considered to be centrally embedded in online communities. The information they share will be more widely distributed and receive more attention, which, in turn, will stimulate them to make more contributions. In addition, patients who aim to increase structural capital have an incentive to share information to develop social interaction ties. Thus, we could conclude that social interaction ties have a positive influence on patients’ information sharing. Therefore, we propose the following hypothesis:

**Hypothesis** **1.** *Patients with high social interaction ties are more likely to share information in online depression communities.*

The relative dimension of social capital refers to those assets created and leveraged through personal relationships [29]. The key aspects of this dimension include strongly identifying with an organization [39], trusting team members [40], and feeling morally obliged to participate in the activities of the organization. Prior research has demonstrated that relational capital is an important asset that benefits both the organization and its members by facilitating the exchange and transfer of information in the communication network of organizations. In this study, we extended the research about relational capital from the organizational level to the individual level and investigated how relational capital will influence patients’ information sharing in online depression communities. We focused on two key facets of the relational dimension: a sense of shared identity and trust, both of which were found in earlier studies to be significantly associated with knowledge sharing in virtual communities.

A sense of shared identity refers to an individual’s sense of belonging to a particular group. Individuals who have a strong sense of identity with the organization define themselves in terms of their group membership and describe themselves as being stereotypical of the group. They feel morally obliged to help others within the organization on the basis of shared membership [34]. In online depression communities, patients with depression share experiences and provide emotional support to each other. They view themselves as members of the community and consider it a duty to assist other patients in improving depression treatment and outcomes. Some recent studies have proven that a sense of identification with the organization will motivate individuals to post valuable information and contribute their knowledge to online virtual communities. Therefore, we believe that in depression communities, patients with a high degree of shared identity are more willing to share information to help other users in the group in the hope of maintaining good relationships with other members. Therefore, we propose the following hypothesis:

**Hypothesis** **2.** *Patients with a strong sense of shared identity are more likely to share information in online depression communities.*

In addition to a sense of shared identity, trust is also an important facet of relational capital that facilitates information sharing in the communication network of organizations [41], as it erases the confusion about whether other members are allies or will act opportunistically [42]. For groups with a high level of trust among members, group members believe that it is worthwhile to contribute knowledge because they believe that contributed knowledge will not be misused or abused. Previous studies have indicated that interpersonal trust among members in an organization can enhance relationship quality to make individual knowledge transfer less costly and to increase the likelihood of knowledge sharing [43]. In online depression communities, patients with depression are able to develop a sense of trust with each other through interaction and communication over a period of time, which promotes their continued intention to share information. In addition, another aspect of trust is to believe that an individual’s efforts will be reciprocated [40]. The purpose of patients participating in online communities is to seek peer support. When participants are expected to share information with the online community, they also expect the mutual reciprocity that justifies their expense in terms of the time and effort they spend contributing their own knowledge. Thus, when community members trust that their knowledge contribution efforts will be reciprocated by other members, they will feel more motivated to make a contribution. In conclusion, we believe that, for online depression communities, when a patient perceives more trust in other community members, he/she is more willing to share information. Therefore, we propose the following hypothesis:

**Hypothesis** **3.** *The patients affected by depression who perceive more trust are more likely to share information in online depression communities.*

The cognitive dimension of social capital refers to resources that provide shared representations, interpretations, and systems of meaning among parties [29]. Cognitive capital posits that members in a group share common understandings and values and have a sense of common purpose [44]. Previous studies have argued that cognitive capital could facilitate the exchanging and sharing of information among members since people usually prefer to interact with those who hold attitudes, opinions, and characteristics that are similar to their own [45,46]. In this study, we focused on two key facets of cognitive capital, expertise and a sense of shared values, and discussed how cognitive capital will influence patients’ information sharing in online depression communities.

Online depression communities offer new opportunities to communicate and seek support for various concerns within a network of patients with different cultural backgrounds and expertise. However, information provided in online communities seems inevitably more difficult to comprehend than that provided through verbal communication [47]. The contributors need high levels of expertise and mastery of the shared language of the community to better understand the community and convey information more effectively and accurately. In other words, even if a patient is expected to exchange and share information to help others suffering from depression, contribution behavior cannot occur unless he/she has the requisite expertise. This argument is supported by several studies reporting that individuals in online virtual communities are reluctant to share their information if they feel they lack sufficient expertise [48]. Therefore, we conclude that personally owned expertise (i.e., knowledge, skills and abilities) helps patients with depression to better understand shared information and enhances their capability in contributing to the online communities, thereby increasing the likelihood of them sharing information. We thus propose the following hypothesis:

**Hypothesis** **4.** *Patients with a high level of expertise are more likely to share information in online depression communities.*

Shared values refer to the common beliefs, norms, interests, and culture of organizational members, describing the extent to which goals and values held by the exchange parties are consistent or compatible [49]. Prior studies suggested that individuals with high shared values can better understand the potential value of exchanging and combining their resources [44], which helps enhance the willingness of individuals to share knowledge in organizations. Online depression communities are composed of patients who share and discuss common interests and goals. The common goals of improving depression outcomes can help patients recognize the importance of information exchange, avoid possible misunderstandings in their communication with one another, and thus provide more opportunities to freely exchange their ideas about or experience with depression detection and treatment. There is evidence that patients with depression are more willing to share information to help those members who hold common values. Moreover, when members perceive that an online community has created an atmosphere in which members are expected to contribute, they often tend to make more contributions to conform to the expectations of other members with shared values. We thus propose the following hypothesis:

**Hypothesis** **5.** *Patients with a strong sense of shared values are more likely to share information in online depression communities.*

This study not only attempts to understand the effect of social capital on information sharing in online depression communities, but it is also concerned with the issue of whether social capital has a different effect on information sharing for those patients who have spent different lengths of time participating in online depression communities. Some previous studies have attempted to classify information sharing into start-sharing and continued-sharing and found that the determinants of continued-sharing decisions are different from those of start-sharing decisions [50]. For new members who have recently joined an online community, some external factors could play an important role in motivating them to start sharing information. Once a user decides to start sharing, however, some internal factors (i.e., those from within the network that cannot be observed without joining) will carry more weight in changing the user’s decision of whether to continue sharing. Some users are likely to stop sharing information if they have bad experiences in participating in community activities. Therefore, we believe that patients with depression will gradually change their perception of online communities as they take more time to participate in the communities, resulting in some dimensions of social capital playing various important roles in their information-sharing decisions. We thus propose the following hypothesis:

**Hypothesis** **6.** *There are significant differences concerning the effect of social capital on information sharing for patients who have spent different lengths of time participating in online depression communities.*

## 2. Materials and Methods

### 2.1. Research Context

We chose a popular online depression community, SunForum, as our data source. The site provides efficient communication channels and convenient information-sharing services where patients can obtain information related to depression and share their experiences about depression detection and treatment. The site also provides a platform for emotional support where some emotional expressions are presented, such as venting, sending positive energy, and showing compassion or empathy, as it is important, especially for patients with major depression, to receive emotional support and encouragement from fellow patients to help alleviate symptoms of depression.

The online community is open to the public, and any patients suffering from depression have access to the website to browse and search the messages posted by community members. Only registered users are allowed to initiate a new topic and reply to other posts in a discussion thread after logging in.

### 2.2. Data Collection

In our study, the available data for empirical analysis have been collected from the online depression community. After over ten years of development, the site has 10,843 registered members who generated a total of 954,537 posts. Among them, 197 active members who had written more than 50 posts each were finally selected in our study. They accounted for 1.84% of all users. The necessary attributes of the posts were stored, including the author, post title, post body content, thread to which the post belonged, and timestamp indicating the date and time of the post. Note that we only used information that was available to the public. We never used any user identification data. Personal information, such as names and ID numbers, was not used or reported as part of the results of the study. Therefore, this study did not raise any ethical or legal concerns. We finally obtained a dataset consisting of 31,440 posts from 197 members for our empirical analysis.

Next, we performed a content analysis to distinguish information-sharing posts from all the posts in our sample. We automatically identified the posts related to knowledge and information sharing by using the search terms “take drugs”, “psychiatric drugs”, and some other depression domain-specific terminologies. Additionally, two postgraduate students with long-term participation in this research field were selected as annotators to manually annotate the posts and classify them into information-sharing posts and other kinds of posts. The interannotator agreement kappa value was 0.874, indicating substantial agreement between the annotators. After deleting the controversial posts from the experimental data, we finally obtained 8591 posts related to information sharing.

### 2.3. Method

We employed panel data regression analyses to investigate which factors had a significant impact on motivating users to share. To test all of the hypotheses, we developed an empirical model with information sharing as the dependent variable and five factors, including social interaction ties, a sense of shared identity, trust, expertise, and a sense of shared values, as independent variables, as shown in Equation (1).

(1) $$Info\_sharing_{i,t+1}=\beta_{0}+\beta_{1}Ties_{i,t}+\beta_{2}Identity_{i,t}+\beta_{3}Trust_{i,t}+\beta_{4}Expertise_{i,t}+\beta_{5}Values_{i,t}+\varepsilon_{i,t}$$

Table 1 provides a summary of all variables. The dependent variable in our empirical analysis, *Info_sharing*_*i*,*t*+1_, was measured as a continuous variable representing the number of posts written by patient *i* in period *t* + 1.

The independent variables in our empirical model were all measures of three social capital dimensions (cognitive, structural, and relational) related to information sharing. First, we employed social interaction ties as the indicator of the structural dimension of social capital and used the variable *Ties_i,t_* to represent the number of patients who interacted with patient i in period *t* to measure social interaction ties between patient *i* and other patients. Second, we chose a sense of shared identity and trust as two key facets of the relational dimension of social capital. The sense of shared identity refers to an individual’s sense of belonging to a particular group and can be reflected through his/her contributions to the group. Thus, we used the variable *Identity_i,t_* to represent the number of posts written by patient *i* in period *t* to measure the sense of shared identity of patient *i*. Previous studies have shown that when parties trust each other, they are more willing to engage in cooperative activity through which further trust may be generated [51]. In online depression communities, peer patients frequently interact with each other through replying to other posts to discuss issues of concern or express emotional support, which further enhances mutual trust. Thus, we used the variable *Trust_i,t_* to represent the number of replies to posts written by patient *i* from other patients in period *t* to measure the perceived trust received by patient *i*. Third, we chose expertise and a sense of shared values as two key facets of the cognitive dimension of social capital. Expertise reflects an individual’s knowledge, skills, and experience. Those patients with long-term struggles with depression have first-hand experience of the disease with all the medical, psychological, and social problems involved and are aware of how the disease can be prevented. The practical information and personal experiences about symptoms and treatments are considered to be particularly valuable for newly diagnosed patients who lack personal experience and seek practical advice. Thus, we used the variable *Expertise_i,t_* to represent the number of posts about knowledge and experience sharing written by patient *i* in period *t* to measure the level of expertise of patient *i*. The sense of shared values refers to the sense of sharing common beliefs, norms, and interests with other organizational members. Members with a high level of shared values can better understand their peer patients and positively reply to the posts concerning depression knowledge introduction and treatment experience sharing from other members. Thus, we used the variable *Values_i,t_* to represent the number of replies to posts about knowledge and experience sharing written by patient i in period *t* to measure the sense of shared values perceived by patient *i*.

## 3. Results

### 3.1. Summary Statistics

Table 2 presents descriptive statistics of the independent variables measuring three social capital dimensions related to information sharing that were used in the study. During the experimental period, each patient in the sample interacted with 642 other patients on average. The average number of posts written by each patient was 158, and the average number of replies to each post was 1584. Each patient, on average, wrote 21 posts about his/her knowledge and experience of depression and received an average of 45 replies from other members.

### 3.2. Correlation Analysis

Table 3 presents a correlation matrix for all measured variables. As seen from the correlation matrix, the dependent variable *Info_sharing* was significantly correlated to all independent variables. This preliminary result provides initial support for our hypotheses.

In addition, we found that there was a significantly positive correlation among all the independent variables. For example, the independent variable *Ties* was positively correlated with *Identity* and *Trust.* It is reasonably well understood that when a patient has a strong sense of shared identity within an online community, he/she has more trust in community members and is more willing to form strong ties with others in the community. The independent variable *Expertise* was positively correlated with *Ties* and *Trust*, indicating that those patients with higher expertise will be more popular in the community and that they are likely to receive more trust and develop stronger social interaction ties. The independent variable *Values* was positively correlated with *Identity* and *Trust*, indicating that shared values help patients understand each other, build trust among individuals, and enhance their sense of belonging to the community.

### 3.3. Regression Analysis

Table 4 presents the results of panel data regression analyses for information sharing. The results showed that the F-value of 14,966.25 was significant (*p* < 0.01), indicating that the overall fit of the regression model was statistically significant at this level. A *t*-test was used in the study to test whether the independent variables had significant effects on the dependent variable. As indicated in Table 4, the independent variables measuring three dimensions of social capital were all significant predictors of information sharing. The detailed analysis is as follows.

First, we sought to evaluate whether the structural dimension of social capital could have a significant effect on information sharing. The results showed that the coefficient of the independent variable *Ties* was significantly positive (B = 0.0348, *p* < 0.01), indicating that social interaction ties had positive effects on information sharing. Thus, Hypothesis 1 is supported.

Second, we examined whether the relational dimension of social capital could have a significant impact on information sharing. The results showed that the coefficient of the independent variable *Identity* was significantly positive (B = 0.1998, *p* < 0.01), indicating that the sense of shared identity had a positive effect on information sharing. Thus, Hypothesis 2 is supported. Similarly, the coefficient of the independent variable *Trust* was significantly positive (B = 0.0054, *p* < 0.01), indicating that interpersonal trust had a positive effect on information sharing. Thus, Hypothesis 3 is supported.

Third, we examined whether the cognitive dimension of social capital could have a significant effect on information sharing. The results showed that the coefficient of the independent variable *Expertise* was significantly positive (B = 0.1072, *p* < 0.01), indicating that a high expertise level had a positive effect on information sharing. Thus, Hypothesis 4 is supported. Similarly, the coefficient of the independent variable *Values* was significantly positive (B = 0.0121, *p* < 0.01), indicating that having a strong sense of shared values had a positive effect on information sharing. Thus, Hypothesis 5 is supported.

We further examined whether there were any differences concerning the effect of social capital on information sharing for patients who spent different lengths of time participating in online depression communities. We first divided the full sample into four subsamples according to duration of online participation. The members who had been online for less than 100 h were categorized as newcomers. The members who had been online between 100 and 300 h were categorized as junior members. The members who had been online between 300 and 800 h were categorized as intermediate members. The members who had been online for more than 800 h were categorized as senior members. Next, we ran the same panel data regression on the four subsamples separately. Then, we performed a comparative analysis of the regression results for the full sample (presented as model 1) and four subsamples (presented as models 2, 3, 4, and 5).

As seen in Table 5, we found that the coefficients of three independent variables *Ties, Identity,* and *Trust* in models 2, 3, 4, and 5 were all significant and had the same signs as the coefficients in model 1. The results further support our tests of Hypotheses 1–3. Moreover, we found that the coefficient of *Ties* in the four models were close to each other, showing that there may be no significant difference in the influence of the social interaction ties on information sharing between different user groups. However, some contrasting results can be found in the coefficient values of *Trust*, which were different in four models and increased as the online participation time increases. We can conclude that trust had a more positive role in promoting information sharing for newcomers than it did for long-term participants in online depression communities, and the effect of trust on information sharing gradually weakened as the online participation time grew.

We then examined whether the two dimensions of cognitive capital, expertise and a sense of shared values, had different effects on information sharing in different user groups. As shown in Table 5, the coefficients of the independent variables *Expertise* and *Values* in model 2 and model 3 were both not significant at the 0.05 level, indicating that for the newcomers and junior members with less online participation time, a high expertise level and a sense of shared values were not significant indicators of their information sharing. In contrast, the coefficients of the independent variables *Expertise* and *Values* in model 5 were both significant, which was in accord with the results observed in the full sample. From the results above, we can see that there were some significant differences concerning the effects of three dimensions of social capital on information sharing for patients who have spent different lengths of time participating in online depression communities. Thus, Hypothesis 6 is supported.

## 4. Discussion

### 4.1. Principal Findings

First, the results of this study support the argument that social capital theory is particularly well suited to explain the paradox of why patients affected by depression voluntarily share information in the online community without receiving any immediate benefits, which is in accord with the findings of previous studies on other types of online communities.

We categorized social capital into three dimensions: structural, relational, and cognitive. We further subdivided these into five subdimensions. The results show that all five factors had significant effects on the information sharing of patients with depression. First, social interaction ties had a positive influence on information sharing. The findings support the idea that patients with strong social interaction ties are considered to be centrally embedded in online depression communities and will receive widespread attention, which will stimulate them to make more contributions. This result is in accordance with the empirical findings in previous research [26]. Second, a sense of shared identity had a positive effect on information sharing. One reasonable explanation for this is that patients with a high sense of shared identity are more likely to feel morally obliged to help others and are more willing to share information in the hope of maintaining good relations with other members. This finding was supported by empirical results in several previous studies [34]. Third, interpersonal trust had a positive effect on information sharing. Results are in accordance with the previous work done by Nonaka [43], proposing that interpersonal trust among members in an organization can enhance relationship quality and increase the likelihood of knowledge sharing. Patients with depression who perceive more trust believe that the information they share will not be misused or abused and their contribution efforts will be reciprocated by other members; therefore, they will consider it worthwhile to share their knowledge and experience. Fourth, a high expertise level had a positive effect on information sharing. It is well understood that patients with a high level of expertise can better understand the community and convey information more effectively and accurately than those with a low level of expertise, thereby increasing the likelihood of the former sharing information. These results are in accordance with the conclusion drawn by Wasko and Faraj [48]. Finally, a strong sense of shared values had a positive effect on information sharing, as indicated by the finding [44] that individuals with high shared values can better understand the potential value of exchanging information, which helps enhance the willingness of individuals to share knowledge. When the patients in online depression communities perceive that other community members hold the same values that they themselves do, they are more willing to share information to help others.

We further divided the full sample into four subsamples to examine whether there was any difference concerning the effect of social capital on information sharing for patients who spent different lengths of time participating in online depression communities. The results showed that for newcomers and junior members with less online participation time, high expertise levels and a sense of shared values were not significant indicators of their information sharing. A possible reason for this is that it is difficult for newcomers to increase their expertise in a very short period of time. Additionally, they will take a considerable amount of time to assess whether there are shared values in the online community. This argument is also supported by the result that, for long-term participants in the online community, expertise and a sense of shared values both have a significant, positive role in promoting information sharing.

### 4.2. Implications

This article makes key theoretical contributions on two fronts. First, our research contributes to previous research by adopting social capital theory to explain what drives patients affected by depression to share information in online communities. Prior research has suggested that social capital theory provides a powerful basis for understanding the paradox of why members in online communities voluntarily share information without receiving any immediate benefits. However, few studies have focused on the field of the online health community to explore whether social capital theory is well suited to explain why patients voluntarily share information in the online health community, let alone in the context of an online depression community. In this study, we extended prior studies and developed a theoretical model proposing that three dimensions of social capital had significant effects on the information sharing of patients with depression. Five research hypotheses were proposed and were also supported by empirical data. Although the sample data are from a Chinese online depression community, we believe that the findings are generalizable to broader populations, such as patient populations of various cultures. The findings help shed light on some theoretical propositions and empirical results obtained in prior work.

Second, our research extends previous theoretical work by considering the effect of the length of online participation time on the information sharing of patients with depression, which was not considered in previous studies. We divided the full sample into four groups according to patients’ online participation time. The empirical results revealed that there was a significant difference concerning the effect of social capital on information sharing for four groups of patients who have spent different lengths of time participating in online depression communities.

Our results have important practical implications for designers and managers of online depression communities. First, this study seeks to address the issues of concern to practitioners—that is, what drives patients affected by depression to share information in the online community? Our empirical results help community managers better understand that nonmonetary benefits, such as social capital, rather than some extrinsic benefits, such as monetary rewards, may be the main drivers of the information sharing of patients with depression. Community managers should focus on how to help information sharers obtain more social capital instead of giving them more extrinsic benefits.

Second, the results revealed that the five factors identified in the study had positive effects on the information sharing of patients with depression. Thus, online community designers and managers should take specific measures and provide humanized support functions to facilitate community members’ access to the five types of social capital resources. For example, the designers and managers might consider improving communication to facilitate patients’ abilities to develop social interaction ties (e.g., developing specific functions for “friends”) and enhance their sense of having a shared identity with the online community (e.g., giving honor points to active users), encourage them to increase mutual trust (e.g., developing more supporting functions such as “likes”, “shares”, and “follows”), offer them more opportunities to increase their expertise (e.g., initiating some learning activities such as online quizzes), and create a welcoming atmosphere in which members are willing to contribute their knowledge and experience to conform to the expectations of other members with shared values.

Third, managers must be aware of the fact that social capital has different effects on information sharing for patients who have spent different lengths of time participating in online depression communities. Thus, community managers could develop differentiated policies for either facilitating the information sharing of newcomers or for encouraging senior members to continue sharing. For example, because it is difficult for newcomers to increase their expertise level in a very short period of time, and they take a considerable amount of time to assess whether they have shared values with other members in online communities, community managers should put an emphasis on learning how to encourage newcomers to strengthen social interaction ties and increase their interpersonal trust rather than trying to increase the newcomers’ expertise or enhance their sense of shared values.

## 5. Conclusions

Online depression communities have become increasingly recognized as a promising platform for communication about depression; they are a place where patients with depression can share information and exchange social support to relieve their depression. However, little is known about what drives patients affected by depression to voluntarily share information online. This article attempted to use social capital theory to explain why community members are willing to invest so much time and effort in sharing their knowledge about and experiences with depression without receiving any economic benefits. Following the theoretical framework of social capital, this study categorized social capital into three dimensions—structural, relational, and cognitive—and proposed six hypotheses to explore how various factors affect patients’ information sharing. We then employed panel data regression analyses to test all hypotheses. The results revealed that (i) all five factors, including social interaction ties, a sense of shared identity, trust, expertise, and a sense of shared values, had significant, positive effects on the information sharing of patients with depression, and (ii) there was a significant difference concerning the effect of social capital on information sharing for patients who spent different lengths of time participating in online depression communities. Our findings have important theoretical implications for research in the area of online depression communities and practical implications for designers and managers of online depression communities.

This study has some limitations. First, we did not use demographic variables in our empirical model due to limited data availability. According to prior studies, demographic characteristics such as gender, age, and marital status can play an important role in information sharing. These additional variables may be incorporated into the models in further studies. Second, we identified five social capital factors in the study that had positive effects on the information sharing of patients with depression. However, there are still some other factors that play important roles in information sharing but were not uncovered in our study. Many explicit or implicit benefits of information sharing are outside the control of online communities and were not available for our study because we only had access to online behavioral data and focused on how online activities affected information sharing. As future work, we intend to perform further analyses of online behavioral data, such as employing text mining methods to analyze the content of online posts and their mood, to develop multiple indicators to measure the variables of interest in this study. Finally, we are limited to the data retrieved from a popular online community for depression in China. However, the online community is just one of many forms of social media. More data from other forms of social media, such as blogs and microblogs, are needed to test whether the results are consistent with our findings. In addition, we will consider whether there are significant differences concerning the effect of social capital on information sharing for patients with various cultures to ascertain the generalizability of our findings in further studies.

## Figures and Tables

**Table 1:** Summary of all variables.

Variables	Description
Dependent variable	
*Info_sharing*_*i*,*t*+1_	The number of posts written by patient *i* in period *t* + 1
Independent variables	
*Ties_i,t_*	The number of patients who interacted with patient *i* in period *t*
*Identity_i,t_*	The number of posts written by patient *i* in period *t*
*Trust_i,t_*	The number of replies to posts written by patient *i* from other patients in period *t*
*Expertise_i,t_*	The number of posts about knowledge and experience sharing written by patient *i* in period *t*
*Values_i,t_*	The number of replies to posts about knowledge and experience sharing written by patient *i* in period *t*

**Table 2:** Descriptive statistics of the independent variables.

Variable	Min	Max	Mean	SD.
*Ties*	3	5261	642	778.94
*Identity*	50	1907	158	191.08
*Trust*	4	29,972	1584	2642.02
*Expertise*	0	622	21	55.91
*Values*	0	2159	45	174.82

**Table 3:** Correlation of the variables.

Variable	*Info_sharing*	*Ties*	*Identity*	*Trust*	*Expertise*	*Values*
*Info_sharing*	1.0000					
*Ties*	0.1702 *	1.0000				
*Identity*	0.2485 *	0.3265 *	1.0000			
*Trust*	0.1624 *	0.6444 *	0.3324 *	1.0000		
*Expertise*	0.0918 *	0.1225 *	0.2328 *	0.1432 *	1.0000	
*Values*	0.0511 *	0.1763 *	0.0834 *	0.1557 *	0.2698 *	1.0000

* Correlation is significant at the 0.05 level (two-tailed).

**Table 4:** Panel data regression analysis.

Variables		Coef.	Std. Err.	*t*	*p* > | *t* |
Structural	*Ties*	0.0348 ***	0.0006	53.92	0.000
Relational	*Identity*	0.1998 ***	0.0010	193.75	0.000
	*Trust*	0.0054 ***	0.0002	29.28	0.000
Cognitive	*Expertise*	0.1072 ***	0.0039	27.63	0.000
	*Values*	0.0121 ***	0.0016	7.76	0.000
	_cons	0.0175 ***	0.0002	53.64	0.000
Model Evaluation					
	Number of observations	1,045,770			
	Number of groups	197			
	R-squared	0.0668			
	F(5, 1045568)	14,966.25 ***			

Notes: *** *p* < 0.01, ** *p* < 0.05, * *p* < 0.1.

**Table 5:** Comparison of regressions on information sharing for different groups.

Variables		Coefficients (SE)			
		Model 2 New	Model 3 Junior	Model 4 Intermediate	Model 5 Senior
Structural	*Ties*	0.0342 *** (0.0026)	0.0230 *** (0.0018)	0.0297 *** (0.0014)	0.0307 *** (0.0011)
Relational	*Identity*	0.1569 *** (0.0021)	0.2412 *** (0.0022)	0.1053 *** (0.0021)	0.2368 *** (0.0020)
	*Trust*	0.0212 *** (0.0012)	0.0138 *** (0.0008)	0.0114 *** (0.0005)	0.0038 *** (0.0003)
Cognitive	*Expertise*	−0.0042 (0.0221)	0.0686 (0.0123)	0.1104 *** (0.0076)	0.1107 *** (0.0062)
	*Values*	0.0016 (0.0075)	−0.0062 * (0.0035)	−0.0103 *** (0.0029)	0.0196 *** (0.0026)
	_cons	0.0121 *** (0.0006)	0.0110 *** (0.0005)	0.0152 *** (0.0005)	0.0305 *** (0.0009)
Model Evaluation					
	Number of observations	25,5791	255,791	266,449	271,778
	Number of groups	48	48	50	51
	R-squared	0.0414	0.0883	0.0394	0.0869
	F-value	2208.09 ***	4954.33 ***	2186.83 ***	5172.53 ***

Notes: *** *p* < 0.01, ** *p* < 0.05, * *p* < 0.1.
